# Electrochemical Sensing of Vanillin Based on Fluorine-Doped Reduced Graphene Oxide Decorated with Gold Nanoparticles

**DOI:** 10.3390/foods11101448

**Published:** 2022-05-17

**Authors:** Venkatesh S. Manikandan, Emmanuel Boateng, Sharmila Durairaj, Aicheng Chen

**Affiliations:** 1 Electrochemical Technology Centre, Department of Chemistry, University of Guelph, 50 Stone Road E, Guelph, ON N1G 2W1, Canada; vmanikan@lakeheadu.ca (V.S.M.); eboate01@uoguelph.ca (E.B.); sduraira@uoguelph.ca (S.D.); 2 Department of Chemistry, Lakehead University, 955 Oliver Road, Thunder Bay, ON P7B 5E1, Canada

**Keywords:** vanillin, electrochemical sensor, reduced graphene oxide, Au nanoparticles, differential pulse voltammetry

## Abstract

4-hydroxy-3-methoxybenzaldehyde (vanillin) is a biophenol compound that is relatively abundant in the world’s most popular flavoring ingredient, natural vanilla. As a powerful antioxidant chemical with beneficial antimicrobial properties, vanillin is not only used as a flavoring agent in food, beverages, perfumery, and pharmaceutical products, it may also be employed as a food-preserving agent, and to fight against yeast and molds. The widespread use of vanilla in major industries warrants the need to develop simple and cost-effective strategies for the quantitative determination of its major component, vanillin. Herein, we explore the applications of a selective and sensitive electrochemical sensor (Au electrodeposited on a fluorine-doped reduced-graphene-oxide-modified glassy-carbon electrode (Au/F-rGO/GCE)) for the detection of vanillin. The electrochemical performance and analytical capabilities of this novel electrochemical sensor were investigated using electrochemical techniques including cyclic voltammetry and differential pulse voltammetry. The excellent sensitivity, selectivity, and reproducibility of the proposed electrochemical sensor may be attributed to the high conductivity and surface area of the formed nanocomposite. The high performance of the sensor developed in the present study was further demonstrated with real-sample analysis.

## 1. Introduction

Vanillin, also known as 4-hydroxy-3-methoxybenzaldehyde, is an essential chemical component of natural vanilla. Vanillin is frequently being used as a flavoring agent in food, beverages, sweets, and other pharmaceutical formulations due to its desirable aroma and flavoring qualities. It also exhibits essential antioxidative and antimicrobial properties against the growth of yeasts, molds, and bacteria [1,2,3]. Furthermore, vanillin has been shown to reduce the risk of cardiovascular disease in humans by preventing the oxidation of low-density lipoproteins (LDL), as well as to reverse the consequences of sickle-cell anemia [4]. On the other hand, significant consumption of synthetic vanillin has been shown to cause severe liver damage and kidney failure [5,6]. Thus, it is of great importance to develop a selective, sensitive, and efficient method for the detection of vanillin.

Currently, many conventional analytical methods, such as ultraviolet (UV)–visible (VIS) spectroscopy, gas (GC) and high-pressure liquid chromatography (HPLC) are utilized for the quantification of vanillin in food and beverages [1,7,8,9]. These analytical methods are known for their accuracy; however, their extremely high operational costs, time-consuming pre-treatment of samples, and requirement of skilled personnel to operate are major drawbacks. In contrast, the electrochemical sensor presents itself as an ideal replacement for the detection of vanillin, as vanillin is an electroactive compound and it can thus be detected by studying its oxidation via facile electrochemical techniques [10,11]. However, the electrochemical detection of vanillin using a bare glassy-carbon electrode (GCE) is challenging, as the oxidation of its molecules occurs at higher positive potentials; reproducibility may also be affected due to potential electrode fouling [12,13]. Thus, the modification of the GCE surface with a good electrocatalyst is desirable to increase the electrochemical response for the oxidation of vanillin.

Several studies have focused on the quantitative measurement of vanillin using various types of nanostructured materials in different buffer media [14,15,16,17,18]. Yardim and co-workers reported on a vanillin-detection method based on a boron-doped diamond electrode using an adsorptive stripping voltammetry methodology [19]. Additionally, Zheng et al. biosynthesized Au–Ag alloy-based nanoparticles (NPs) decorated on a GCE surface for the detection of vanillin. When compared to the unmodified GCE, the sensor displayed a better electrochemical performance [20]. Silva and colleagues reported on the development of a nanosensor for the detection of vanillin based on Au NPs stabilized with poly (allylamine hydrochloride) [21]. Those studies have shown that electrochemical techniques are promising for the detection of vanillin; however, further improvement is required for practical applications.

The use of noble metals coupled with carbon-based electrochemical sensors may enhance the performance of electrochemical sensors for detecting and quantifying vanillin [22,23,24,25]. Graphene and its derivatives are widely used in a variety of applications due to its excellent physicochemical properties [26,27,28,29,30]. High electron mobility and a large specific surface area make them suitable for the development of high-performance electrochemical sensors, as graphene-based nanomaterials may facilitate the electron transport between the electrode surface and the target analytes [31,32,33]. Due to their high electrical conductivity, high specific surface area and outstanding electrocatalytic capabilities, Au NPs are also frequently found in graphene-based nanocomposites [34,35,36].

At the molecular and atomic levels, doping graphene with heteroatoms such as fluorine, chlorine, nitrogen, boron, and sulfur may improve its electrochemical behavior and electrocatalytic activities, while altering its electronic and physicochemical properties, as well as those of its derivatives [37,38,39,40]. Fluorine doping in carbon-based materials has generated great interest as the difference in the electronegativity of fluorine and carbon may amend the physicochemical features of carbon-based materials [41,42]. Shahzad and co-workers developed a multifunctional electrically tunable F-rGO composite. Compared to the undoped reduced graphene oxide (rGO), the F-rGO sample exhibited a better catalytic ability, where the fluorine atoms served as catalytic active sites due to their high electronegativity [43]. An et al. synthesized a F-rGO nanocomposite with a significant presence of CF_2_ groups in the graphene matrix for lithium-ion batteries. The authors attributed the excellent cycling stability of their developed F-rGO to the vacancies, defects and the highly stable and electrochemically inert CF_2_ groups formed in the graphene matrix [44].

Herein, we report on the development of the Au/F-rGO/GCE-based electrochemical sensor for vanillin detection. The sensor was prepared by drop-casting a fluorine-doped graphene-oxide (F-GO) solution on a GC electrode, followed by the electrochemical reduction to form F-rGO and the electrodeposition of Au to make the Au/F-rGO/GCE before its use. The electrochemical and analytical capabilities of this novel electrochemical sensor were studied using various electrochemical methods including cyclic voltammetry (CV), linear sweep voltammetry (LSV), and differential pulse voltammetry (DPV). The fabricated electrochemical sensor demonstrated excellent sensitivity, stability, and reproducibility.

## 2. Materials and Methods

### 2.1. Materials

Vanillin (4-hydroxy-3-methoxybenzaldehyde), potassium chloride (KCl), potassium ferricyanide (K_3_[Fe(CN)_6_]), potassium nitrate (KNO_3_), chloroauric acid (HAuCl_4_), and other reagents were received from Sigma-Aldrich Canada Co (Oakville, ON, Canada). All solutions used for the experiments were obtained using an ultrapure water obtained from Nanopure Diamond UV water purification system (18.2 MΩ·cm). All experiments were conducted in a 0.1 M phosphate-buffered-saline (PBS), sulfuric-acid (0.1 M, H_2_SO_4_), and sodium-hydroxide (NaOH, 0.5 M, pH 7.0) solutions.

### 2.2. Electrochemical Sensor Fabrication

An improved Hummers’ method was utilized to synthesized F-GO. Initially, a mixture of 1.0 g graphite, 90.0 mL sulfuric acid, 10.0 mL orthophosphoric acid (H_3_PO_4_), and 20.0 mL hydrofluoric acid (HF) was prepared. The mixture was then vigorously stirred for two hours under constant temperature of 50 °C. Afterwards, 4.5 g potassium permanganate (KMnO_4_) was slowly added to the initial mixture and continuously stirred for an additional 15 h. Next, the prepared mixture was added to a different prepared mixture of 5.0 mL 30% hydrogen peroxide (H_2_O_2_) and 100.0 mL purified ice. The final product, F-GO solution, was then centrifuged at 4000 rpm for a period of 15 min followed by washing and rinsing with 30% HCl acid, then purified water, ethanol, and finally diethyl ether. The suspended F-GO material was dried at 50 °C overnight in an oven.

A 2.0 mg/mL F-GO solution (with water) was prepared, which was then sonicated for 1 h. In the preparation of the electrode, a GCE was initially polished with 1.0 and 0.05 μm alumina slurries, after which the polished electrode was sonicated in water for 5 min. The prepared F-GO solution was drop-casted onto the clean GCE and left to air dry for 4 h to obtain a F-GO/GCE. The as-prepared F-GO/GCE was then electrochemically reduced in 0.1 M PBS solution to obtain F-rGO/GCE. Furthermore, electrodeposition of Au NPs onto the as-prepared F-rGO/GCE electrode to obtain Au/F-rGO/GCE was performed in a mixture of 2.0 mM HAuCl_4_ and 0.1 M KNO_3_ solution. For comparison, Au NPs were electrodeposited onto a bare GCE (Au/GCE) under the same condition.

### 2.3. Physicochemical Characterization and Electrochemical Techniques

FE-SEM images were obtained using a FEI Quanta FEG 250, whereas X-ray diffraction (XRD) characterization was carried out using a PANalytical Empyrean powder diffractometer, with a Cu Kα (λ = 1.5405 Å) radiation source. A CHI potentiostat (CHI-660D, CH Instruments, Austin, TX, USA) was used for the electrochemical studies. All CV, LSV and DPV experiments were performed in a conventional three-electrode electrochemical system, where the Au/F-rGO/GCE, a platinum wire and a standard Ag/AgCl electrode were used as the working, counter and reference electrodes, respectively. Prior to each experiment, the solution was purged with pure argon gas for 15 min and the electrochemical cell above the electrolyte continued to purge during the electrochemical measurements.

## 3. Results and Discussion

### 3.1. Surface Characterization

**Figure 1:**
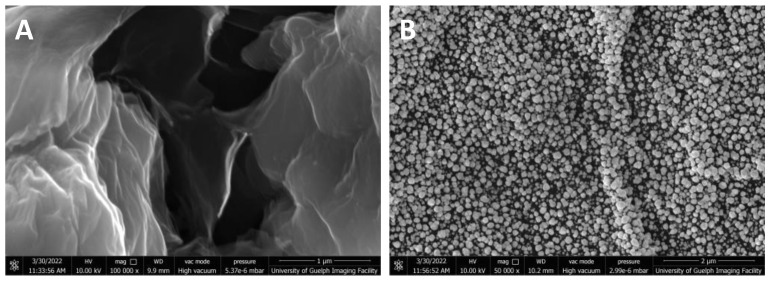
SEM images of (**A**) F-rGO, and (**B**) Au/F-rGO.

The surface morphologies of the as-prepared F-rGO and Au/F-rGO were characterized via FE-SEM (Figure 1), while EDX was used to confirm the presence of Au and fluorine in the presence of the nanocomposite as shown in Figure S1. Figure 1A depicts the SEM image of F-rGO, which exhibited a wrinkled texture with the presence of irregular edges, which is a typical feature of graphene-based nanomaterials. The crumpled surface contributed to the high surface area, which ensured a high surface-contact area and electrocatalytic activity during the electrodeposition of the Au NPs. As seen from the SEM image (Figure 1B) and elemental mappings (Figures S2 and S3), the presence of the electrodeposited Au NPs on the surface of the F-rGO were uniformly distributed. The crystallite structure of the Au NPs was characterized by X-ray diffraction measurements. The XRD diffractograms of the as-synthesized materials are displayed in Figure S4. The F-rGO had no diffraction peaks in the range from 2θ = 36.0° to 46.0°. In contrast, for the Au/F-rGO sample, there were two diffraction peaks at 2θ = 38.4° and 44.6°, which were assigned to Au (111) and Au (200), respectively, showing that the Au NPs had a face-centered cubic (fcc) structure (JCPDS #: 04-0784). Using Scherrer’s formula, the average crystallite size of the electrodeposited Au NPs was calculated to be ~16 nm.

### 3.2. Electrochemical Characterization

**Figure 2:**
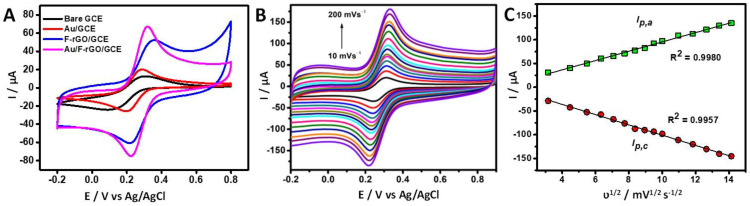
(**A**) Cyclic voltammograms of the response of bare GCE (black), Au/GCE (red curve), F-rGO/GCE (blue curve) and Au/F-rGO/GCE (pink curve) in 0.1 M KCl solution containing 5.0 mM [Fe(CN)_6_]^3−/4−^ at the scan rate of 50 mV/s. (**B**) Cyclic voltammograms of the response of Au/F-rGO/GCE at various scan rates (10–200 mV/s^1^). (**C**) Plots of redox peak current response against the square root of the scan rate.

As shown in Figure 2A, CV was employed to investigate the electrochemical behaviors of the fabricated electrochemical sensors, bare GCE, F-rGO, Au, and Au/F-rGO modified GCEs in the presence of the electrochemical redox mediator, K_3_[Fe(CN)_6_]^3−/4−^. A pair of well-defined redox peaks that corresponded to the oxidation and reduction of [Fe(CN)6]^3−/4−^ were obtained. While the bare GCE and Au/GCE exhibited low redox peak currents, the Au/F-rGO/GCE and F-rGO/GCE exhibited significantly high redox peak currents and a small anodic–cathodic peak-to-peak separation (Δ*Ep*). These results showed that the incorporation of the Au NPs and F-rGO surface enhanced the electrochemically active surface area and the electron-transfer kinetics. Moreover, the estimated anodic-to-cathodic peak-current ratio (*I_p,a_*/*I_p,c_*) of 0.96 confirmed a reversible [Fe(CN)_6_]^3−/4−^ redox process at the Au/F-rGO/GCE and F-rGO/GCE electrodes.

Moreover, both the anodic (*I_p,a_*) and cathodic (*I_p,c_*) peak currents for the redox process at the Au/F-rGO/GCE electrode were noticeably higher than the other electrodes. The enhanced behavior may be attributed to the synergistic effect of the incorporation of fluorine atoms, which are highly electronegative, into the graphene matrix by means of semi-ionic carbon-fluoride (C-F) and the highly conductive Au. Figure 2B presents the CV curves of the Au/F-rGO/GCE recorded at different scan rates ranging from 10–200 mV/s in a KCl–ferricyanide electrolyte solution, showing that the peak currents were increased with the increase in the scan rate. Figure 2C displays the plots of the anodic and cathodic peak current versus the square root of the scan rates; they were fitted using the Randles–Sevick equation:

(1) $$i_{p}=268600n^{3/2}ACD^{1/2}v^{1/2}$$

where *i_p_*, *n*, *A*, *D*, *C*, and *v* represent the peak current (µA), the number of electrons transferred, surface area (cm^2^), the diffusion coefficient (cm^2^/s), concentration of the probe (mol/L), and scan rates (V/s), respectively. The linear relationship confirmed that the redox reactions at the Au/F-rGO/GCE was diffusion-controlled. The electrochemically active surface area (ECSA) of the Au/F-rGO/GCE was calculated to be 0.38 cm^2^, which was much larger that of the bare GCE (0.07 cm^2^), the Au/GCE (0.11 cm^2^) and the F-rGO/GCE (0.24 cm^2^).

### 3.3. Electrochemical Behavior of Vanillin Using CV and DPV

**Figure 3:**
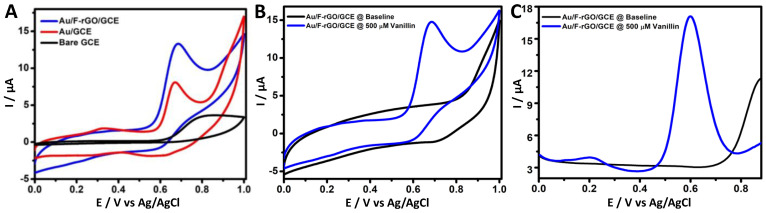
(**A**) Cyclic voltammograms of the bare GCE (black curve), Au/GCE (red curve), and Au/F-rGO/GCE (blue curve) recorded in a 0.1 M PBS solution (pH 7.0) containing 500.0 µM vanillin at a scan rate of 50 mV/s. (**B**) Cyclic voltammograms and (**C**) differential pulse voltammograms of the Au/F-rGO/GCE in a 0.1M PBS solution in the absence (black curve) and in the presence of 500.0 µM vanillin (blue curve).

Figure 3A displays the cyclic voltammograms of bare GCE, AuGCE, and Au/F-rGO/GCE recorded at the scan rate of 50 mV/s in a 0.1 M PBS solution containing 500.0 µM vanillin. The electro-oxidation of vanillin generated a larger anodic peak at the Au/F-rGO/GCE than at the bare GCE and Au/GCE, indicating that the fluorine-doped reduced graphene oxide and the Au NPs played significant role. The *I_p,a_* for the bare GCE, Au/GCE, and Au/F-rGO/GCE was estimated to be 3.74, 8.15, and 13.33 µA, respectively. For comparison, the CV curves of the F-rGO/GCE are presented in Figure S5, showing that the peak current for the vanillin oxidation was much lower than that of the Au/F-rGO/GCE. The higher electrocatalytic activity demonstrated by the fabricated electrochemical sensor, Au/F-rGO/GCE, may be attributed to the synergistic effect between the Au NPs and F-rGO NCs. The peak current obtained from the oxidation of vanillin was approximately four times larger than that of the bare GCE, suggesting that the Au/F-rGO/GCE was efficient for the electro-oxidation of vanillin.

The electro-oxidation of vanillin at the Au/F-rGO/GCE was also investigated using CVs recorded in a 0.1 M PBS solution in the absence (black curve) and presence (blue curve) of 500.0 µM vanillin (Figure 3B), further confirming that the strong peak was due to the electrochemical oxidation of vanillin. In addition, DPV was employed to investigate the oxidation responses of vanillin at the Au/F-rGO/GCE (Figure 3C). A well-defined and strong anodic peak (15.63 µA) was observed at 0.6 V for the electro-oxidation of 500.0 µM vanillin, showing that the DPV technique exhibited an improved sensitivity in contrast to CV. The notable *I_p,a_* values obtained by CV and DPV further confirmed the strong oxidation of vanillin at the Au/F-rGO/GCE.

As seen in Figure 3B, no reduction peak appeared in the reversed scan, revealing that the electrochemical oxidation of vanillin was irreversible. LSV was thus employed to investigate the effect of the scan rate on the electro-oxidation of vanillin. Figure S6A presents a series of the LSV curves of the Au/F-rGO/GCE recorded in a 0.1 M PBS solution (pH 7.0) containing 100.0 µM vanillin. With the increase in the scan rate from 10 to 100 mV s^−1^, the current was increased. Figure S6B displays the plot of the peak current versus the scan rate; the good linear correlation (R^2^ = 0.9972) suggested that the oxidation of vanillin at the Au/F-rGO/GCE electrode was adsorption-controlled.

### 3.4. Optimization of Au-Deposition Time

**Figure 4:**
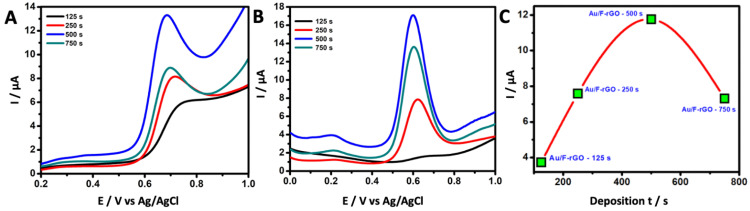
(**A**) Linear sweep voltammogram and (**B**) differential pulse voltammogram of the response of various Au/F-rGO/GCE electrodes (with different Au-deposition times: 125, 250, 500, and 750 s) in 0.1 M PBS solution (pH 7.0) containing 500.0 µM vanillin. (**C**) Relationship of Au-NP-deposition time with respect to the anodic peak current.

Using LSV and DPV techniques, the effects of Au-deposition times on the F-rGO/GCE with respect to the electrochemical oxidation of vanillin were investigated as shown in Figure 4. An amperometric method was used to deposit the Au NPs onto the F-rGO/GCE surface in a 2.0 mM HAuCl_4_ + 0.1 M KNO_3_ electrolyte under the constant potential of −0.4 V (vs. Ag/AgCl). To obtain the optimum deposition time, four different deposition times of 125, 300, 500, and 750 s were chosen with the corresponding formed electrodes denoted as Au/F-rGO/GCE-125 s, Au/F-rGO/GCE-250 s, Au/F-rGO/GCE-500 s, and Au/F-rGO/GCE-750 s, respectively. The peak current for the oxidation of vanillin increased with the increase in the deposition time from 125 to 500 s. As shown in Figure 4C, a further increase in the deposition time from 500 to 750 s resulted in a decrease in the peak current, indicating that 500 s was the optimal deposition time for achieving excellent electrocatalytic performance for the vanillin oxidation at the formed Au/F-rGO/GCE.

### 3.5. Effect of the Vanillin Concentration

**Figure 5:**
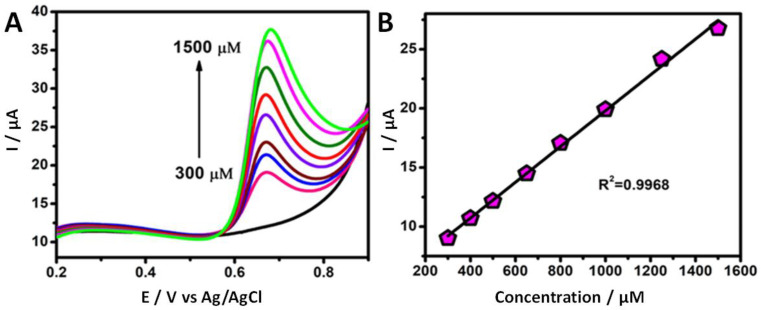
(**A**) Linear sweep voltammogram of the response of Au/F-rGO/GCE in 0.1 M PBS solution (pH 7.0) containing various vanillin concentrations (300.0 to 1500.0 μM). (**B**) Linear relationship between the oxidation of vanillin (anodic peak current) with respect to various concentrations. Scan rate: 50 mV/s.

The electro-oxidation behavior of vanillin at the as-prepared Au/F-rGO/GCE was further studied using LSV in 0.1 M PBS solution (scan rate = 50 mV/s), while varying the vanillin concentrations. It is obvious from Figure 5A that the absence of vanillin (zero analyte concentration) in the electrolyte solution provided no anodic peak, while a sharp response was observed when 300.0 μM analyte was added to the electrolyte solution. As the concentration of vanillin was increased from 300.0 to 1500.0 µM, the *I_p,a_* value for the electro-oxidation of vanillin also increased accordingly. Figure 5B shows the calibration plot obtained for the *I_p,a_* value for the oxidation against the varying vanillin concentrations. According to the linear-regression equation, *I_p,a_* (μA) = 4.671 (μA/μM) * C (μM) + 0.152 μA, a good linear correlation (R^2^ = 0.9968) with respect to the concentration range was achieved.

### 3.6. Analytical Determination of Vanillin

**Figure 6:**
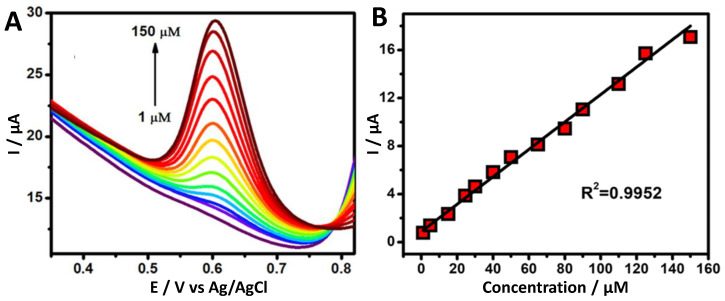
(**A**) Differential pulse voltammograms of Au/F-rGO/GCE in 0.1 M PBS solution (pH 7.0) containing various vanillin concentrations (1.0 to 150.0 μM). (**B**) Linear relationship between the oxidation of vanillin (anodic peak current) with respect to various concentrations. Scan rate: 50 mV s^−1^.

Under the optimized conditions, DPV measurements were employed to investigate the electrochemical oxidation of vanillin at the fabricated electrochemical sensors. Figure 6 depicts the DPV curves of the electrochemical oxidation of various vanillin concentrations at the Au/F-rGO/GCE. It was observed that the anodic peak current was directly proportional to the vanillin concentration. Figure 6B illustrates the linear relationship between the concentration (*C*, µM) and the current (*I_p,a_*, µA) for the electrochemical oxidation of vanillin in the concentration ranges of from 1.0–150.0 μM. The following regression equation is presented as *I_p,a_* (μA) = 0.8772 (μA/μM) * *C* (µM) + 0.114 μA with R^2^ = 0.9952. The limit of detection (LOD), which was calculated as 0.15 μM, was based on three times the standard deviation of the blank divided by the slope,

(2) $$\mathrm{LOD}=\frac{3\sigma }{s}$$

where σ denotes the standard deviation of five blank measurements, and *s* represents the slope obtained from the calibration plot. The limit of quantification (LOQ) was also calculated to be 0.5 μM, obtained via the formula:

(3) $$\mathrm{LOQ}=\frac{10\sigma }{s}$$

where σ denotes the standard deviation of five blank measurements, and *s* represents the slope obtained from the calibration plot. Table S1 compares the performance of the Au/F-rGO/GCE with various vanillin electrochemical sensors reported in the literature, revealing that the sensor developed in the present study exhibited a wide linear range and a low detection limit.

### 3.7. Stability, Reproducibility and Interference Tests as Well as Real-Sample Analysis

The reproducibility of the Au/F-rGO/GCE was tested by preparing four separated electrodes, as shown in Figures S7A,B. The relative-standard-deviation (RSD) value for the *I_p,a_* value measured at the four different electrodes was estimated to be 2.82%, demonstrating a good electrode-to-electrode reproducibility of the Au/F-rGO/GCE sensor. Using the DPV method, 25 successive scans were recorded to investigate the stability of the fabricated electrochemical sensor, as shown in Figure S8. The Au/F-rGO/GCE maintained ~94% of its initial *I_p,a_* response to 500.0 µM vanillin in a 0.1 M PBS solution, indicating good stability and no fouling of the electrochemical sensor during the detection. The selectivity of the Au/F-rGO/GCE was further tested. In the presence of a 2.5 mM of interferents, including glucose, gallic acid, CH_3_COONa, CuSO_4_, MgCl_2_, KCl, and NH_4_NO_3_, over 95.2% retainability of the response to 500 µM vanillin was achieved, showing excellent anti-interference attributes. Moreover, the developed Au/F-rGO/GCE was employed to demonstrate its ability to determine the presence of vanillin in milk samples using the standard calibration analysis with the spiking of two different concentrations. Figure S9 illustrates the real-sample analysis using DPV in the milk samples, which proved the possibility of this developed sensor to be used in food-sample analysis. Two-percent commercial milk was used. A volume of 1.0 mL of the milk was diluted with 9.0 mL of PBS solution, then 10.0 and 50.0 µM vanillin were spiked into the milk solution, respectively. Figure S9 presents the DPV response of the Au/F-rGO/GCE to the spiked 10.0 and 50.0 µM vanillin. As listed in Table S2, the Au/F-rGO/GCE showed a high recovery percentage between 97.1 and 96.8 with a relative standard deviation (RSD) of 3.9% and 3.2%, confirming that the proposed Au/F-rGO/GCE sensor can be used for the electrochemical detection of vanillin in food samples.

## 4. Conclusions

The development of a novel Au/F-rGO/GCE electrochemical sensor was successfully achieved and used for the electrochemical detection of vanillin. Our surface characterization showed that the Au nanoparticles were uniformly deposited on the F-rGO surface. The formed Au/F-rGO nanocomposite exhibited a much larger electrochemically active surface area and faster electron transfer compared to the Au NPs and F-rGO-modified GCE. The results of electrochemical characterization demonstrated that the Au/F-rGO/GCE was efficient at oxidizing vanillin compared to the GCE, Au/GCE and F-rGO/GCE. The developed Au/F-rGO/GCE sensor exhibited satisfactory analytical capability with good linearity over the selected calibration range, a high sensitivity and a low LOD. The sensor also demonstrated high selectivity for the detection of vanillin in the presence of co-existing ions and other possible interferent molecules. Moreover, the Au/F-rGO/GCE exhibited appreciable repeatability, good reproducibility, and possessed excellent chemical stability. Furthermore, the results obtained from the sensor revealed a satisfactory recovery result from real-sample analysis, which confirmed its practical applicability.

## Data Availability

The data presented in this study are available on request from the corresponding author.

## Supplementary Materials

The following supporting information can be downloaded at: https://www.mdpi.com/article/10.3390/foods11101448/s1, Figure S1. EDX spectrum of F-rGO and Au/F-rGO; Figure S2. Elemental mapping images of F-rGO with red, green, and blue dots representing carbon, oxygen, and fluorine elements, respectively (scale bare = 10 μm); Figure S3. Elemental mapping images of Au/F-rGO with red, green, blue, and yellow dots representing carbon, oxygen, fluorine, and gold elements, respectively (scale bare = 10 μm); Figure S4. X-ray diffractogram of F-rGO and Au/F-rGO; Figure S5. Cyclic voltammograms of F-rGO/GCE in a 0.1 M PBS solution (pH 7.0) in the absence (black curve) and in the presence of 500.0 µM vanillin (blue curve); Figure S6. (A) Linear sweep voltammogram response of Au/F-rGO/GCE in 0.1 M PBS solution (pH 7.0) containing 100.0 μM vanillin at various scan rate (10–100 mV s^−1^). (B) Linear relationship between the anodic peak current and the scan rate; Figure S7. (A) Linear sweep voltammogram response of four different Au/F-rGO/GCE electrodes in 0.1 M PBS solution (pH 7.0) containing 500.0 μM vanillin at a scan rate of 50 mV s^−1^. (B) Estimated anodic peak current from individual electrode; Figure S8. Consecutive linear sweep voltammogram scans of Au/F-rGO/GCE in 0.1 M PBS solution (pH 7.0) containing 500.0 μM vanillin at a scan rate of 50 mV s^−1^; Figure S9. DPV response for the 10 and 50 μM vanillin in milk samples in a PBS solution (pH 7.0). Table S1. Comparison of the performance of the Au/F-rGO/GCE with the vanillin electrochemical sensors recently reported in the literature; Table S2. Recovery tests by spiking vanillin into a milk solution. References [45,46,47,48,49,50] are cited in the Supplementary Materials.
