# Supplementary material for: Electrochemical Sensing of Vanillin Based on Fluorine-Doped Reduced Graphene Oxide Decorated with Gold Nanoparticles
Source: Foods. 2022 May 17;11(10):1448. doi: 10.3390/foods11101448 (PMC9140755; doi:10.3390/foods11101448)
Supplement: Supplementary file 1 [file foods-11-01448-s001.zip › foods-1686130-supplementary.pdf]

Supporting Information

Electrochemical sensing of vanillin based on fluorine-doped reduced graphene oxide decorated with gold nanoparticles

Venkatesh S. Manikandan[1,2], *Emmanuel Boateng*[1], *Sharmila Durairaj*[1] and *Aicheng Chen*[1,*]

[1] Electrochemical Technology Centre, Department of Chemistry, University of Guelph, 50 Stone Road E, Guelph, Ontario N1G 2W1, Canada

[2] Department of Chemistry, Lakehead University, 955 Oliver Road, Thunder Bay, Ontario P7B 5E1, Canada

[*] E-mail : aicheng@uguelph.ca

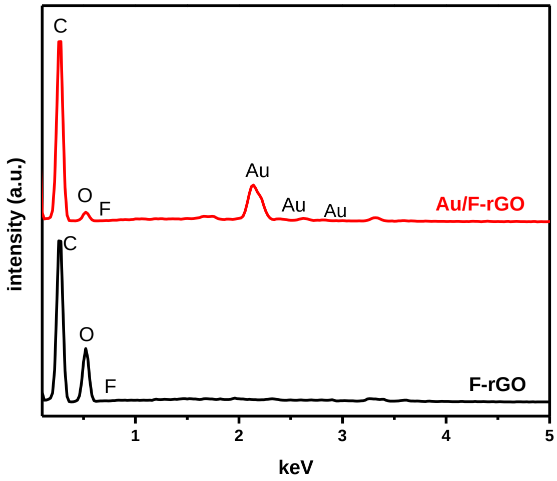

Figure S1. EDX spectrum of F-rGO and Au/F-rGO.

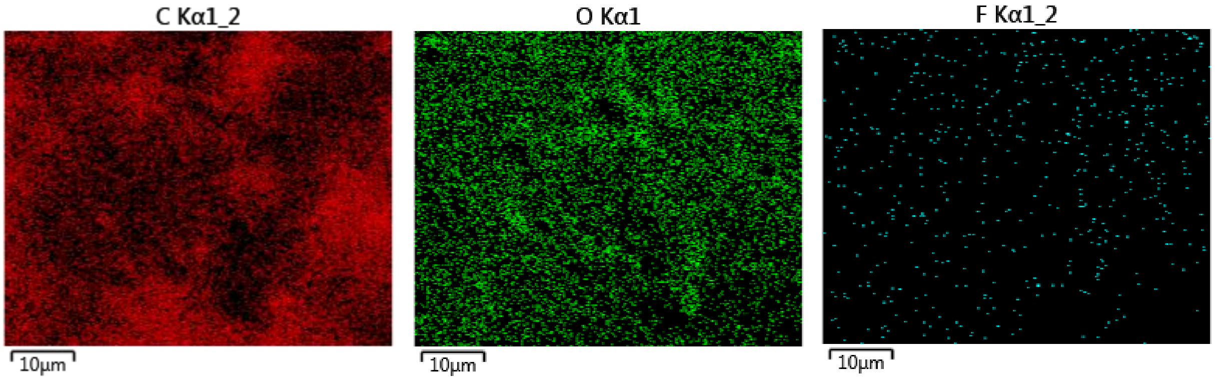

Figure S2. Elemental mapping images for F-rGO with red, green, and blue dots representing carbon, oxygen, and fluorine elements, respectively (scale bare = 10 μm).

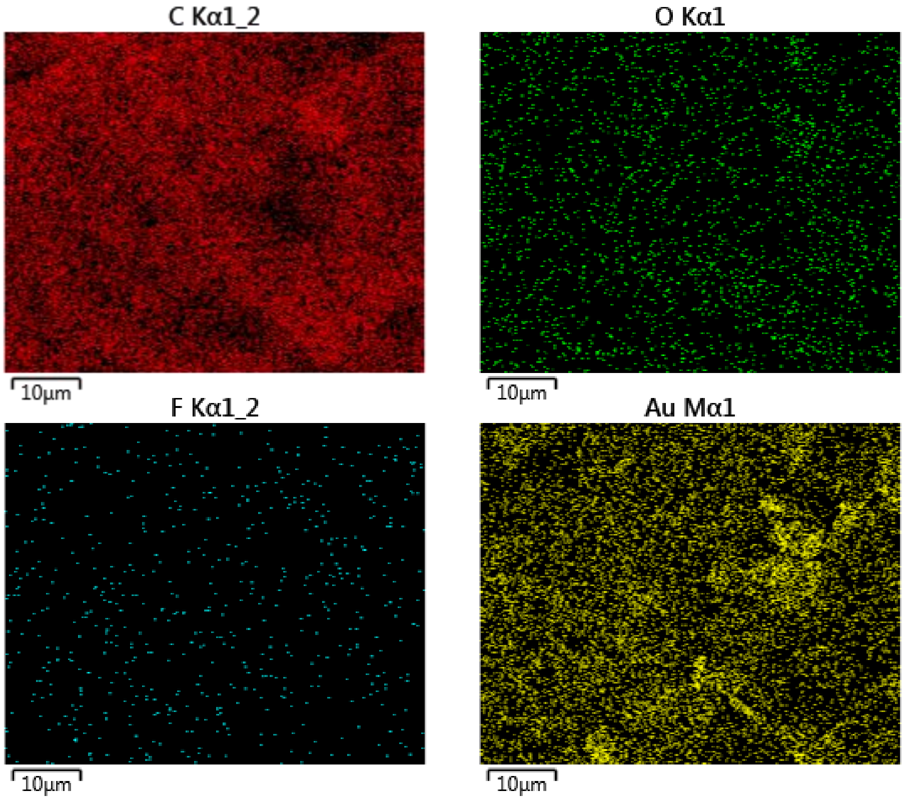

Figure S3. Elemental mapping images for Au/F-rGO with red, green, blue, and yellow dots representing carbon, oxygen, fluorine, and gold elements, respectively (scale bare = 10 µm). It can be observed that the Au atoms are mostly uniformly dispersed on the surface of the F-rGO support.

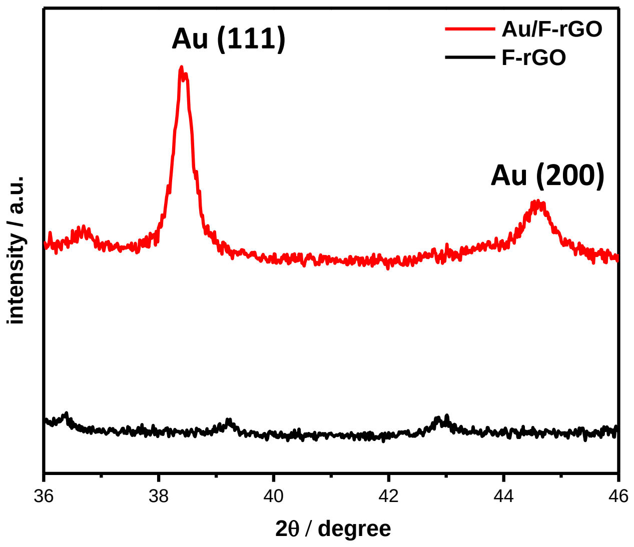

Figure S4: X-ray diffractogram of F-rGO and Au/F-rGO.

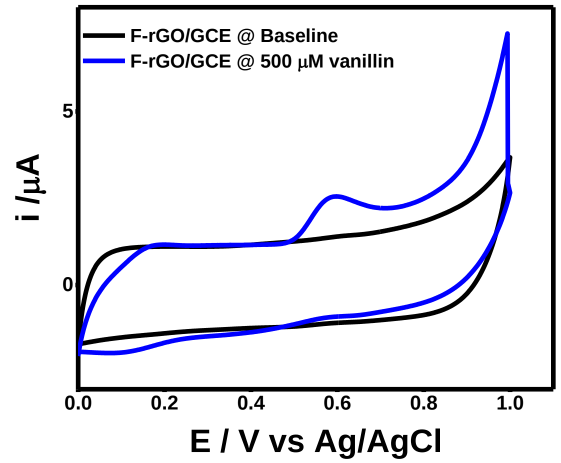

Figure S5. Cyclic voltammograms of F-rGO/GCE in a 0.1 M PBS solution (pH 7.0) in the absence (black curve) and in the presence of 500.0 µM vanillin (blue curve) at a scan rate of 50 mVs^{-1}.

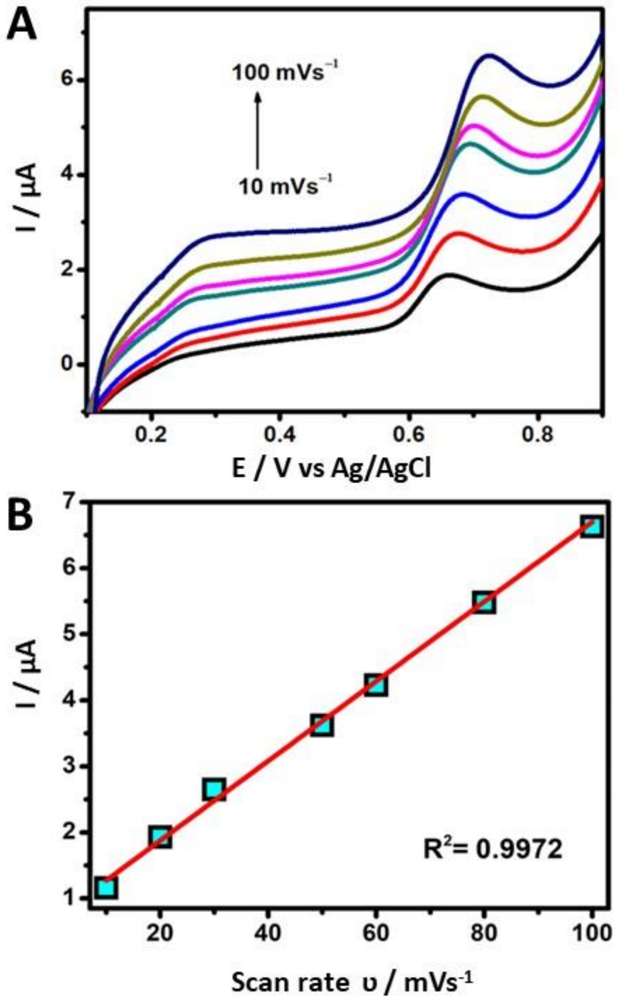

Figure S6. (A) Linear sweep voltammogram response of Au/F-rGO/GCE in 0.1 M PBS solution (pH 7.0) containing 100.0 µM vanillin at various scan rate (10 – 100 mV s^{-1}). (B) Linear relationship between the anodic peak current and the scan rate.

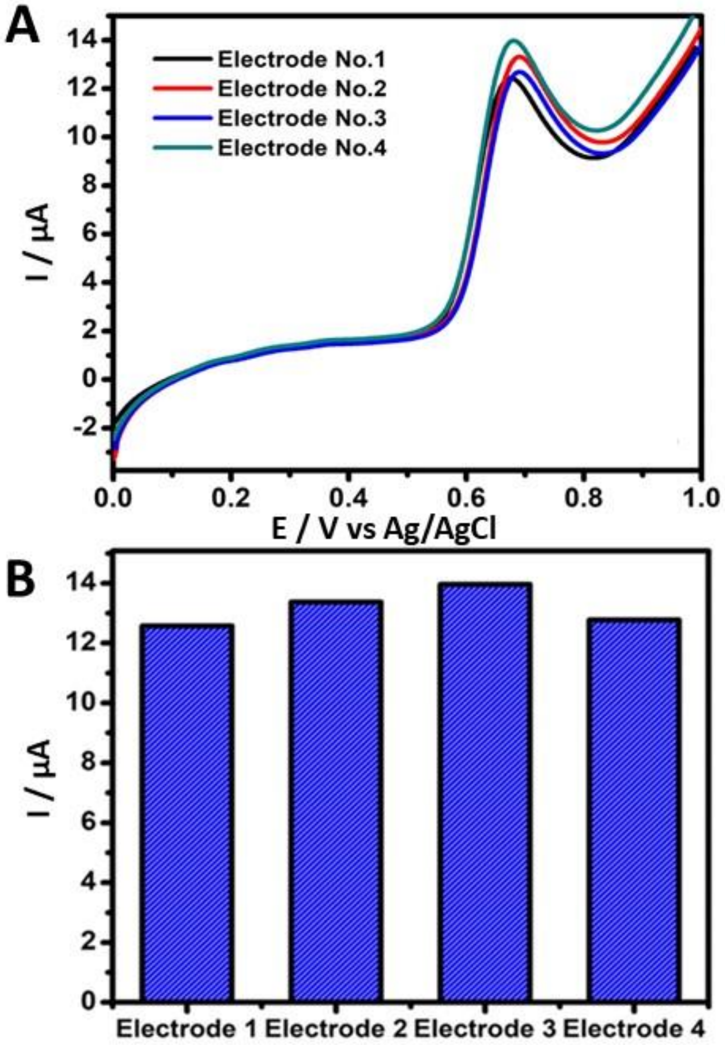

Figure S7. (A) Linear sweep voltammogram response of four different Au/F-rGO/GCE electrodes in 0.1 M PBS solution (pH 7.0) containing 500.0 µM vanillin at a scan rate of 50 mV s^{-1}. (B) Estimated anodic peak current from individual electrode.

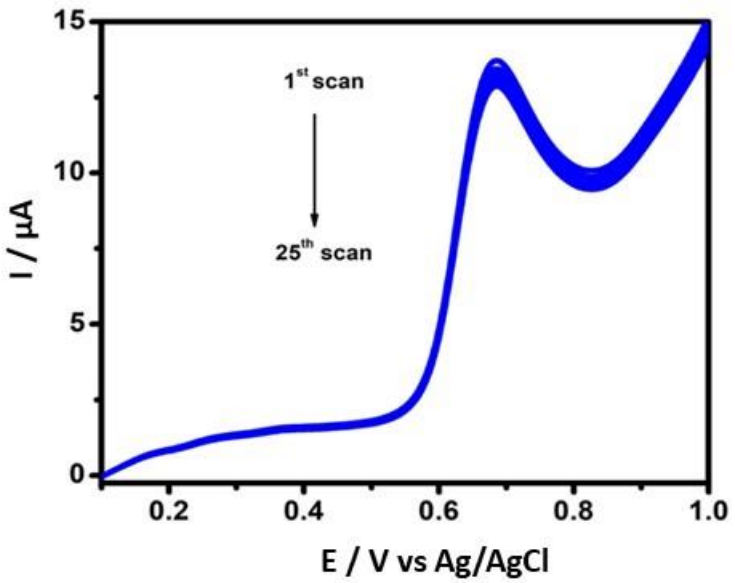

Figure S8. Consecutive linear sweep voltammogram scans of Au/F-rGO/GCE in 0.1 M PBS solution (pH 7.0) containing 500.0 µM vanillin at a scan rate of 50 mV s^{-1}.

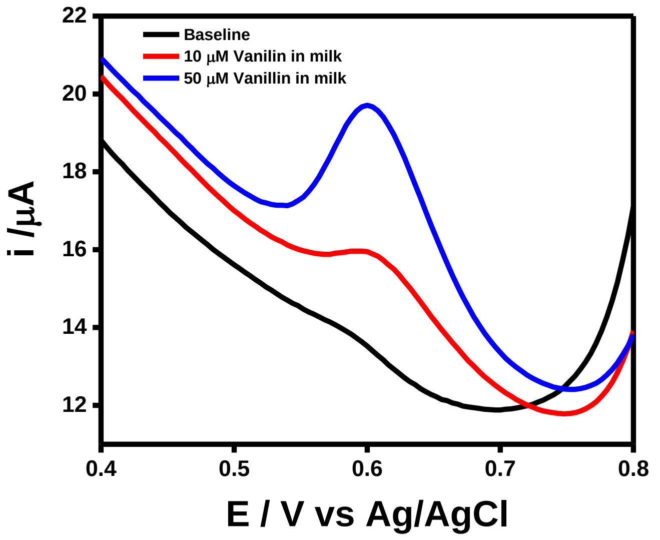

Figure S9. DPV response for the 10 and 50 μM vanillin in milk samples in a PBS solution (pH 7.0).

Table S1. Comparison of the performance of the Au/F-rGO/GCE with the vanillin electrochemical sensors recently reported in the literature.

Electrode	Method	Linear range [µM]	LOD [µM]	Ref.
CNT – SPE	DPV	2.5 – 750	1.03	1
CTABMGPE	DPV	4 - 15	1.29	2
Graphene film-coated GCE	DPV	20 -70	1.29	3
ATT film	DPV	1.1 – 76.4	0.19	4
TBAC-900/GCE	DPV	5 - 1150	0.68	5
PMOMGPE	DPV	10 – 35	0.07	6
**Au/F-rGO/GCE**	**DPV**	**1 - 150**	**0.15**	**Present work**

CNT – SPE = carbon nanotube screen-printed electrode; CTABMGPE = CTAB modified graphene paste electrode; ATT = 3-amino-1,2,4-triazole-5-thiol/Au; TBAC = Cajeput tree bark derived activated carbon; PMOMGPE = Poly(methyl orange) modified graphene paste electrode

Table S2. Recovery tests by spiking vanillin into a milk solution.

**Analyte**	**Added (µM)**	**Found (µM)**	**Recovery (%)**	**RSD (%)**
**Milk**	10.0	9.7	97.1	3.9
	50.0	48.4	96.8	3.2

Reference:

1. Chen, L.; Chaisiwamongkhol, K.; Chen, Y.; Compton, R. G. Rapid electrochemical detection of vanillin in natural vanilla. *Electroanal.* **2019**, 31, 1067-1074.
2. Raril, C.; Manjunatha, J.G. A simple approach for the electrochemical determination of vanillin at ionic surfactant modified graphene paste electrode. *Microchem. J.* **2020**, 154, 104575.
3. Peng, J.; Hou, C.; Hu, X. A graphene-based electrochemical sensor for sensitive detection of vanillin. *Int. J. Electrochem. Sci.* **2012**,7, 1724 – 1733
4. Calam, T.T.; Uzun, D. Rapid and selective determination of vanillin in the presence of caffeine, its electrochemical behavior on an Au electrode electropolymerized with 3-Amino-1,2,4-triazole-5-thiol. *Electroanal.* **2019**, 31, 2347-2358.
5. Veeramani, V.; Madhu, R.; Chen, S.-M.; Veerakumar, P.; Syu, J.-J.; Liu, S.-B. Cajeput tree bark derived activated carbon for the practical electrochemical detection of vanillin. *New J. Chem.* **2015**, 39, 9109 – 9119.
6. Monnappa, A.B.; Manjunatha, J.G.G.; Bhatt, A.S.; Nagarajappa, H. Sensitive and selective electrochemical detection of vanillin at graphene-based poly (methyl orange) modified electrode. *J. Sci.: Adv. Mater. Devices* **2021**, 6, 415-424
